# A Clinical Evaluation of the GemStar® and the AmbIT® Pumps for Patient-Controlled Epidural Analgesia

**DOI:** 10.5812/aapm.7513

**Published:** 2012-09-13

**Authors:** Aneeta Sinha, Michael Paech, Rupert Ledger, Nolan McDonnell, Elizabeth Nathan

**Affiliations:** 1 Department of Anaesthesia, Queen Alexandra Hospital, Portsmouth, UK; 2 School of Medicine and Pharmacology, The University of Western Australia, Perth, Australia; 3 Department of Anaesthesia and Pain Medicine, King Edward Memorial Hospital for Women, Perth, Australia; 4 Women and Infants Research Foundation, Perth, Australia

**Keywords:** Analgesia, Epidural, Analgesia, Patient-Controlled, Analgesia, Obstetrical, Equipment and Supplies, Infusion Pumps

## Abstract

**Background:**

Patient-controlled analgesia is used for both labor and postoperative analgesia.

**Objectives:**

This study aimed to assess user satisfaction and functionality of two ambulatory, electronic patient controlled analgesia devices, the GemStar pump Hospira Inc., Illinois, USA) and the ambIT Ambulatory Infusion Therapy pump (Sorenson Medical Products, Utah, USA).

**Patients and Methods:**

It was a randomized clinical trial of laboring women and postoperative gynecology patients receiving patient-controlled epidural analgesia. Patients were randomized to use one of the pumps and both anesthesiologists and patients completed questionnaires about aspects of pump function, and rated their satisfaction with the equipment. Midwives and high-dependency unit nurses also evaluated the pumps in each clinical setting.

**Results:**

Forty patients, 20 laboring women and 20 postoperative patients were randomized and completed the study. The pumps were compared by nine anesthesiologists. Patient and staff satisfaction with both devices was high. Patient satisfaction did not significantly differ between groups (median 10 [8, 10] for the GemStar and 10 [9, 10] for the ambIT, P = 0.525]. The median staff satisfaction score was 8 [6, 8] for the GemStar and 7 [5, 8] for the ambIT (P = 0.154). Both patient cohorts rated each pump highly for most aspects of clinical function. Staff rated the ambIT pump more favourably with respect to portability and storage at the bedside whilst the GemStar had better assessments with respect to its consumables and interactions involving the electronic interface.

**Conclusions:**

Both devices were well-rated by patients and staff, with no significant difference between them for overall satisfaction, and only minor differences with respect to their respective strengths and weaknesses.

## 1. Background

There are many electronic and single-use patient controlled analgesia (PCA) devices available that enable drug administration via intravenous, epidural or other routes (1-9). In general, electronic pumps provide more flexible programming options, such as background continuous infusion at different rates and multiple options for the demand bolus dose and the lockout interval. Patient controlled epidural analgesia (PCEA) for labor and postoperative pain management is safe and effective (1, 10). An electronic pump used for PCEA in our institution for many years is the GemStar® pump (Hospira Inc., Illinois, and USA). The ambIT® Ambulatory Infusion Therapy pump (Sorenson Medical Products, Utah, and USA) is also of appropriate design for PCEA and was recently approved for the Australian market. Both devices are compact, portable, ambulatory-style PCA pumps that provide continuous or intermittent analgesic solution delivery by means of easy-to-program interfaces, and they also provide multiple programming and alarm functions. The GemStar pump has more than one power source, whereas the ambIT pump is a lightweight, battery-operated device designed for optimum portability. User satisfaction and functionality of these pumps were evaluated in the clinical practice settings of PCEA for labor or postoperative analgesia. Patients and relevant health care providers, namely anesthesiologists, nurses and midwives, evaluated the pumps.

## 2. Objectives

The primary objective of the study was to compare user-satisfaction. The null hypothesis was that there would be no significant difference between the GemStar and the ambIT pump in the primary outcomes of the combined staff satisfaction score and the combined patient satisfaction score.

## 3. Patients and Methods

This randomized clinical trial was conducted at a single, tertiary-referral hospital. Institutional ethical approval was sought for the study but was deemed to be unnecessary by the Ethics Committee of the Women and Newborn Health Service. The study was registered with the hospital Product Evaluation committee. Questionnaires (available on request) were developed by the authors to assess the clinical functionality and perceived safety of the GemStar and ambIT PCEA pumps. The two pumps (Figure) were compared on different occasions, in different patients, who had consented to PCEA for either labor analgesia or postoperative analgesia after major gynecological surgery. For both patient populations, the anesthesiologists preparing the pumps (after sealed envelope allocation based on a computer-derived randomization sequence) and the patients were asked to complete questionnaires. Additionally, in the labor cohort, midwives participated, and in the postoperative cohort, high-dependency unit nurses participated. Exclusion criteria were age < 18 or > 75 years; American Society of Anesthesiologists (ASA) score > 3; illicit drug use or opioid dependence. Multiparous laboring women were excluded if the cervical dilatation was > 4 cm, if delivery appeared impending or if fetal abnormality was present. Demographic data recorded were patient age, height, weight, body mass index, gravidity, parity, cervical dilatation and duration of pump use.

**Figure:**
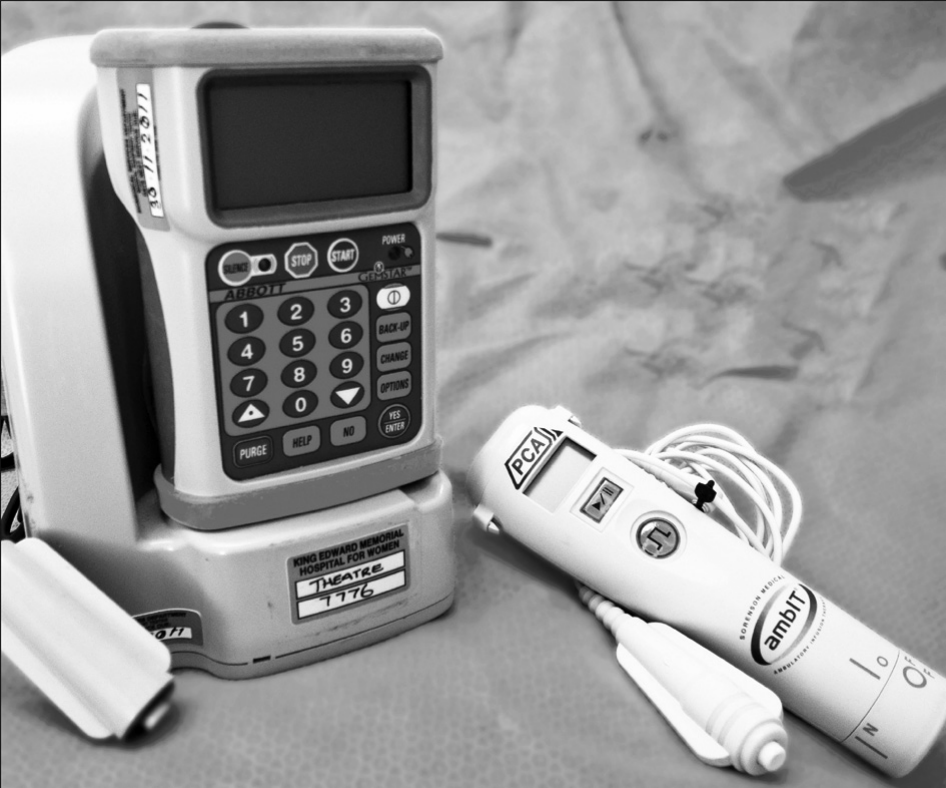
Photo of the GemStar® (Left) and the ambIT® (Right) Pumps

The PCEA solution delivered was standardized according to hospital protocols, including an infusion at 6 mL/h for labor analgesia and 4 mL/h for postoperative analgesia, and 5 mL on-demand with a 15 minute lockout for both. The participating anesthesiologist was responsible for programming the pump and subsequent program changes were the responsibility of the duty anesthesiologist. Each participating staff member assessed at least one sample of both pumps, but patients were only exposed to one pump. Prior to the study, all staff attended instructional sessions, received standard manufacturer information and had the opportunity to familiarize themselves with both devices. Patient questionnaires were completed at the end of clinical use and were collected by a research nurse not involved in the patient’s care. Staff completed the same questionnaires With the exception of the question about pump programming after their final exposure to the pumps, and the questionnaires were returned by internal mail or collected by a research nurse, although the questionnaire was available from the outset, to enable prospective reflection. The dual primary endpoints of the study were the 0-10 numerical rating scores for patient satisfaction, and staff satisfaction with the pumps (0 being not satisfied and 10 being completely satisfied). The staff score was the averaged combined score of all participating staff (i.e. anesthesiologists, nurses and midwives). The secondary endpoints included satisfaction and the personal preference of each group of staff (anesthesiologists, nurses, or midwives) and ratings of a number of utilities such as pump assembly, programming, ease of use, function and information display. The latter were assessed largely by means of 10-point numerical scales, bordered by appropriate extremes of opinion. Multiple questionnaires completed by medical or nursing staff were analysed as independent data on a per patient basis. In order to detect a reduction in mean satisfaction score from 9 (SD 1) to 8 (alpha 0.05, power 80%) (7), a power analysis indicated that 20 pumps of each type would need to be assessed (Power Analysis and Sample Size (PASS) Statistical Software, 2008). Descriptive statistics were summarized using medians, 25^th^ and 75^th^ percentiles. Demographic continuous data and numerical rating scores were analysed by the independent-samples Mann-Whitney U-test and categorical data by the Fisher Exact test. SPSS 18.0 statistical software (SPSS Inc, Chicago, IL) was employed for data analysis. All hypothesis tests were two-sided and the level of significance was P < 0.05.

## 4. Results

Forty patients, 20 laboring women and 20 postoperative patients completed the study. Pumps were programmed and evaluated by nine anaesthetists. Among each patient cohort, 10 patients were randomized to use a GemStar pump and 10 to an ambIT pump. No patient required re-allocation to a different pump. The demographic data of patient groups within each cohort were similar (Tables 1 and 2). Patient and staff satisfaction with both devices was high. Patient satisfaction did not significantly differ between the groups (median 10 [8-10] for the GemStar pump and 10 [9, 10] for the ambIT pump, P = 0.525). The median staff satisfaction score was 8 [6, 8] for the GemStar pump and 7 [5, 8] for the ambIT pump (P = 0.154).

**Table 1:** Demographic Data of the Postoperative Cohort a

	No. (Median) b	No. (Median) c
Age, y	58 (52-60.5)	63 (50.5-67.3)
Height, m	1.64 (1.60-1.67)	1.61 (1.57-1.71)
Weight, kg	67.5 (54-92)	74 (62.3-83.3)
Body mass index, kg/m^2^	29 (22.5-37)	27 (26-28.8)

^a^P = NS

^b^GemStar (25^th^-75^th^ percentile)

^c^ambIT (25^th^-75^th^ percentile)

**Table 2:** Demographic Data of the Labor Cohort a

	No. (Median) ^b^	No. (Median) ^c^
Age, y	20 (24.3-34.8)	31 (28.8-32)
Height, m	1.67 (1.59-1.73)	1.65 (1.63-1.72)
Weight, kg	87.5 (72.5-109.5)	100.5 (87-116.3)
Body mass index, kg/m^2^	34 (28-38)	36 (31-42.8)
Gravidity	2.5 (2-4.5)	3 (1-3)
Parity	1 (0-2.5)	0.5 (0-1.25)
Cervical dilation, cm	2 (0-2)	2 (1-3)
Duration of pump use, h	5.5 (4.1-9.4)	6.0 (4.7-12.4)

^a^P = NS

^b^GemStar (25^th^-75^th^ percentile)

^c^ambIT (25th-75th percentile)

The patient responses are shown in Table 3. There were no significant differences between the pumps for any parameter assessed. Both cohorts of patients rated both pumps highly for most aspects of clinical function, with median scores of 9 or 10. The GemStar pump received a lower median score (6.5 (0.25, 10) vs. 10 (6, 10)) from laboring women with respect to how bothersome the pump alarm proved to be (0 representing a lot and 10 not at all). The majority of patients were happy to use the pump they had used again, with both pumps in both patient cohorts scoring a median value of 10 (0 representing not at all happy to use again and 10 very happy to use again). The staff responses are shown in Table 4. There were no significant differences in satisfaction score for the pumps among any sub-set of staff (data not shown). For labor analgesia, four of nine anesthesiologists preferred the GemStar, four the ambIT pump and one was undecided. For postoperative analgesia, five preferred the GemStar, two the ambIT and two were undecided. In comparison to the GemStar pump, staff considered the ambIT pump to be more portable (P < 0.001) and its weight more acceptable (P < 0.001). Anesthesiologists rated the ambIT pump as easier to store at the bedside (P < 0.05). Both medical and nursing staff found the disposables of the GemStar pump easier to assemble than those of the ambIT pump (P < 0.05) and nursing staff found it easier to change the epidural solution reservoir of the GemStar pump than that of the ambIT pump (P < 0.001). With respect to the electronic interface, anesthesiologists reported that it was easier to see and follow instructions on the GemStar pump display screen, compared with the ambIT pump (P < 0.05). They rated the GemStar pump easier to program (P < 0.001) and the nursing staff favored the GemStar pump for monitoring patient information (P < 0.05). The pump ratings did not significantly differ for various alarm functions, but nursing staff found the GemStar pump alarm information clearer and pump security functions easier to use (P < 0.001 and P < 0.05 respectively).

**Table 3:** Patient Questionnaire Responses

	Postoperative Cohort (25^th^-75^th^ Percentile), No. (Median)			Laboring Cohort (25^th^-75^th^ Percentile), No. (Median)		
	GemStar, n = 10 a	ambIT, n = 10 a	*P*value	GemStar, n = 10 a	ambIT, n = 10 a	*P*value
**Pump design/portability**						
How easy is it to find the pump button to activate it? (0 very difficult, 10 very easy)	10 (7.25, 10)	10 (8,10)	0.91	10 (9.75, 10)	10 (9.5, 10)	0.91
How easy is it to push the button to activate the pump? (0 very difficult, 10 very easy)	10 (10, 10)	10 (9.5, 10)	0.50	10 (10, 10)	10 (10, 10)	1.00
How restrictive was the pump in terms of access to it? (0 very restrictive, 10 not restrictive at all)	10 (9, 10)	10 (8.5, 10)	0.78	10 (9, 10)	10 (10, 10)	0.32
How easily did the pump and its tubing get tangled or in the way? (0 a great deal, 10 not at all)	9.5 (9, 10)	10 (8.75, 10)	0.85	9 (6.5, 10)	10 (6, 10)	0.72
How easy did you find moving around with the pump? (0 not easy at all, 10 very easy)	10 (7.25, 10)	10 (9, 10)	0.69	10 (7, 10)	10 (7, 10)	0.69
**Pump alarms**						
How much did the alarms bother you? (0 a lot, 10 not at all)	10 (10, 10)	10 (10, 10)	1.00	6.5 (0.25, 10)	10 (6, 10)	0.32
How annoying is the sound of the pump alarm? (0 very annoying, 10 not annoying at all)	10 (10, 10)	10 (9.75, 10)	0.80	10 (0.75, 10)	10 (8, 10)	0.67
**General**						
How comfortable have you been while using the pump? (0 extremely uncomfortable 10 extremely comfortable)	10 (8, 10)	10 (9.75, 10)	0.58	10 (6.5, 10)	9 (6.5, 10)	0.80
How easy was the pump to use? (0 very difficult, 10 very easy)	10 (10, 10)	10 (10, 10)	1.00	10 (10, 10)	10 (9.5, 10)	0.74
Please rate how much you liked the pump? (0 really disliked, 10 really liked)	10 (8, 10)	10 (9, 10)	0.58	9 (8, 10)	10 (9.75, 10)	0.14
If you were to have a similar procedure in the future how happy would you be to use this particular pump again? (0 not at all happy to use again, 10 very happy to use again)	10 (9.75, 10)	10 (9, 10)	0.74	10 (8.5, 10)	10 (9.75, 10)	0.68

^a^n represents both the number of participating patients and the number of questionnaires completed

**Table 4:** Staff Responses a

	Anesthesiologists, No. (Median)			Nurses and Midwives, No. (Median)		
	n = 18 b,c	ambit, n = 19 b	*P *value	n = 18^b^, ^c^	ambIT, n = 19 b	*P* value
**Pump Design/Characteristics**						
How portable is the pump? (0 not portable at all, 10 very portable)	4 (3,7)	8 (7,9)	< 0.001	5 (2.5,8.5)	10 (10, 10)	< 0.001
How acceptable is the weight of the pump? (0 unacceptable, 10 ideal)	5 (3,7)	8 (7,9)	< 0.001	6 (3.5, 8.5)	10 (9,10)	< 0.001
How easy is the pump to store at the bedside? (0 very difficult, 10 very easy)	7 (5,8)	8 (7,8)	0.03	8 (5,10)	9 (8,10)	0.13
How acceptable is the power supply system? (V)	6.5 (4.5,8.25)	8 (7,9)	0.04	8 (3.75, 10)	9.5 (7.25,10)	0.12
How easy is it to see and follow instructions on the display screen? (0 very difficult, 10 very easy)	8 (6.5,9)	6 (4,7)	0.02	8 (6.5,10)	7 (3,9)	0.13
**Pump Programming and Disposables**						
How easy is the pump to program? (0 very complex/difficult, 10 very easy/intuitive)	8 (7,9)	5 (3,7)	< 0.001	-	-	-
How easy is it to assemble the disposables? (0 very complex/difficult, 10 very intuitive/easy)	7 (6.75,9)	6 (5,7)	0.03	7 (5,10)	2 (1.25,7)	0.03
How easy is it to fill / prime the cassette? (0 very complex/difficult, 10 very intuitive/ easy)	7 (6,8)	6 (5,7)	0.11	-	-	-
How easy is it to change the reservoir of epidural solution during use? (0 very easy, 10 very difficult)	7 (5.5,8)	5 (4.25, 7.75)	0.12	9.5 (5.75, 10)	3 (0,7)	< 0.001
How easy is it to change pump program settings during use? (0 very complex/difficult, 10 very intuitive/easy)	8 (5,8.25)	6.5 (5,8)	0.21	-	-	-
**PCA Information**						
How easy is it to monitor patient information about drug use during pump use? (0 very difficult, 10 very easy)	7 (5.5,8)	6 (5,7.25)	0.40	10 (8,10)	6.5 (3,10)	0.02
**Pump Alarms and Security**						
How acceptable is the range of alarm functions? 0 inadequate, 10 ideal	6.5 (5,8)	6 (5,8)	0.75	7 (6,9)	6 (4.5, 8.25)	0.31
How clear is the reason for any alarm activation? (0 not clear, 10 very clear)	7 (4.75,8)	5 (4.25, 5.75)	0.09	8 (6,10)	4.5 (3, 7.25)	<0.001
How easy are the alarms to disable and reset? (0 very complex/difficult, 10 very easy)	5 ( 3.75,7)	6(4.5,7)	0.74	5 (3,9)	4 (3,6.5)	0.43
How easy is it to use the pump security functions? (0 very complex/difficult, 10 very easy)	7 (6,8)	6 (4,7)	0.10	8 (5,10)	5 (2.75, 7.25)	0.03
**From the Patient’s Perspective**						
How simple do you think the pump is for patients/women to use? (0 not simple at all, 10 very simple)	8 (8,9.25)	9 (7,9)	0.54	10 (8.75, 10)	10 (9,10)	0.91
How easy is it to help the patient/woman with a position change or mobilization during use? (0 very difficult, 10 very easy)	-	-	-	8 (6,9)	8 (5.5, 9.25)	0.64
**General**						
How does this pump compare with other electronic PCA pumps you have used? (0 very unfavorable, 10 very favourable)	7 (5, 8)	6 (4,7)	0.09	8 (4.5,9.5)	9 (7,10)	0.11

^a^Blank cells represent questions not asked of that particular staff group

^b^n represents the number of questionnaires completed

^c^GemStar(25^th^-15^th^ percentile)

## 5. Discussion

In this small, randomized, qualitative study no significant difference was found in overall patient or staff satisfaction with the GemStar and the ambIT patient-controlled analgesia pumps, when used for PCEA. Patients reported high levels of satisfaction with both pumps and reported being able to locate, identify and activate the pump button with ease, these being desirable human factor design features of PCA pumps (11). Almost all patients indicated that they were happy to use the same pump again, although an obvious limitation of the study, which was not of ‘cross-over’ design, was that they were only exposed to one of the two pumps. Both anesthesia and nursing staff also reported high satisfaction with both pumps. To allow them direct comparison, it was attempted to expose each staff member to more than one type of each pumps. This was achieved for the participating anesthesiologists but not all midwifery or nursing staff, which was another limitation of the study. The ambIT pump is smaller, lighter, and more portable than the GemStar pump. These are desirable attributes, particularly for ambulant patients. Despite its greater size and weight, the GemStar pump was also rated well in terms of not limiting mobility. With the increasing complexity of PCEA regimens and PCA pump programming, the potential for mis-programming increases. Compared with mechanical devices, electronic pumps have greater potential for medication error, especially excessive drug delivery. In one series nearly 40 per cent of PCA errors involved the delivery of an improper dose (12). Drug overdose is the major safety concern, because even a small programming error can lead to serious patient harm (11). Misprogramming errors leading to adverse patient outcomes appear rare, the estimated incidence falling between 1 in 33,000 and 338,000, although fatal outcomes are likely to be more numerous than reported (13). Many recommendations have been made to enhance the safety of PCA pumps (13). User interfaces are improved if human factors, and engineering techniques are incorporated in the design process (14). The Emergency Care Research Unit (ECRI) has recommended that pumps have advanced error-reduction features, such as bar-coding, dose error reduction systems and computer-based pump programming software that detects and prevents dose-related infusion errors or the programming of incorrect infusion settings (15). The latest ECRI evaluation of nine PCA infusion pumps, which included the GemStar pump but not the ambIT pump, did not recommend the GemStar pump because it lacks dose error reduction systems and safety software (11). The ambIT pump does not incorporate these types of safety features either. With respect to clinical utility, simple measures that facilitate accurate pump programming, data collection and problem solving include possession of a display screen which is large enough to be read easily, and easy-to-navigate menus for the health care provider. The ambIT pump was more popular with respect to storage in the delivery room and the GemStar pump rated more favorably with respect to consumables containing epidural solution and for some interactions involving the electronic interface, probably because it has a larger display screen. Opinion as to which pump was preferable in each of the clinical settings was divided. A potential source of bias was that staff was generally more familiar with the GemStar pump, and that the ambIT pump had not been used previously in the institution. However labor unit staff was not familiar with either pump, because a single-use mechanical PCA device is used in this area. In summary, two ambulatory electronic patient-controlled analgesia pumps were assessed in two cohorts of patients, one in labor and the other in the postoperative period. Both pumps were rated highly by patients and staff, with no significant difference in satisfaction, and only minor differences in terms of their respective strengths and weaknesses.
